# ST-Segment Elevation in Lead aVR With Global ST-Segment Depression: Never Neglect Left Main Coronary Artery (LMCA) Occlusion

**DOI:** 10.7759/cureus.26522

**Published:** 2022-07-03

**Authors:** Ruchita Kabra, Sourya Acharya, Sandeep Kamat, Sunil Kumar

**Affiliations:** 1 Department of Medicine, Jawaharlal Nehru Medical College, Datta Meghe Institute of Medical Sciences (Deemed to be University), Wardha, IND; 2 Cardiology, Topiwala National Medical College & B. Y. L. Nair Charitable Hospital, Mumbai, IND

**Keywords:** global, lead avr, electrocardiogram, mismatch oxygen supply, circumferential subendocardial ischaemia

## Abstract

An electrocardiographic pattern consisting of elevated ST segment in lead aVR with global ST-segment depression in multi-leads indicates circumferential subendocardial ischaemia due to mismatch of oxygen supply and demand. This finding of an echocardiogram is strongly suggestive of occlusion of proximal part of left anterior descending part of coronary artery. We describe a patient who presented to us with complaint of chest pain and had ST-segment elevation in lead aVR and global ST-segment depression in multi-leads and was suspected to have occlusion in left anterior descending artery. Patient was taken for early coronary angiography and angioplasty then. It was found that patient had occlusion in proximal part of of left anterior descending part only. Hence, early recognition of the condition and its prognosis can help preventing complications.

## Introduction

Global depression in ST segment in leads representing inferior and anterolateral areas with elevation in ST segment in lead aVR corresponds with subendocardial ischemia circumferentially, which suggests an injury vector directing towards ventricles. When a patient presenting with chest pain and with ECG findings as above is believed to have occlusion in left main coronary artery (LMCA) or triple-vessel coronary artery disease, the predictive value of above diagnosis is 75% [1]. As per the American Heart Association (AHA), an ECG showing ST depression of more than 0.1 mV in about eight leads and ST elevation in lead aVR should suggest that “ischema occurring because of left main coronary artery or triple-vessel coronary artery obstruction” [1].

Early recognition of this ECG findings correlated with manifestations is important in reverting a bad prognosis in acute LMCA occlusion because early coronary intervention is life saving and can prevent further complications too.

## Case presentation

A 52-year-old woman arrived in the emergency room with a two-hour history of severe chest pain radiating to her neck. For a year, she had episodes of exertional angina pectoris. Hypertension and obesity that were not effectively controlled were risk factors for coronary artery disease. She was already taking oral antihypertensive drug amlodepine 5 mg once a day. Her blood pressure (BP) was 160/95 mmHg. An auscultation of the heart revealed quiet heart sounds with no murmurs. There were no rales on pulmonary auscultation.

**Figure 1:**
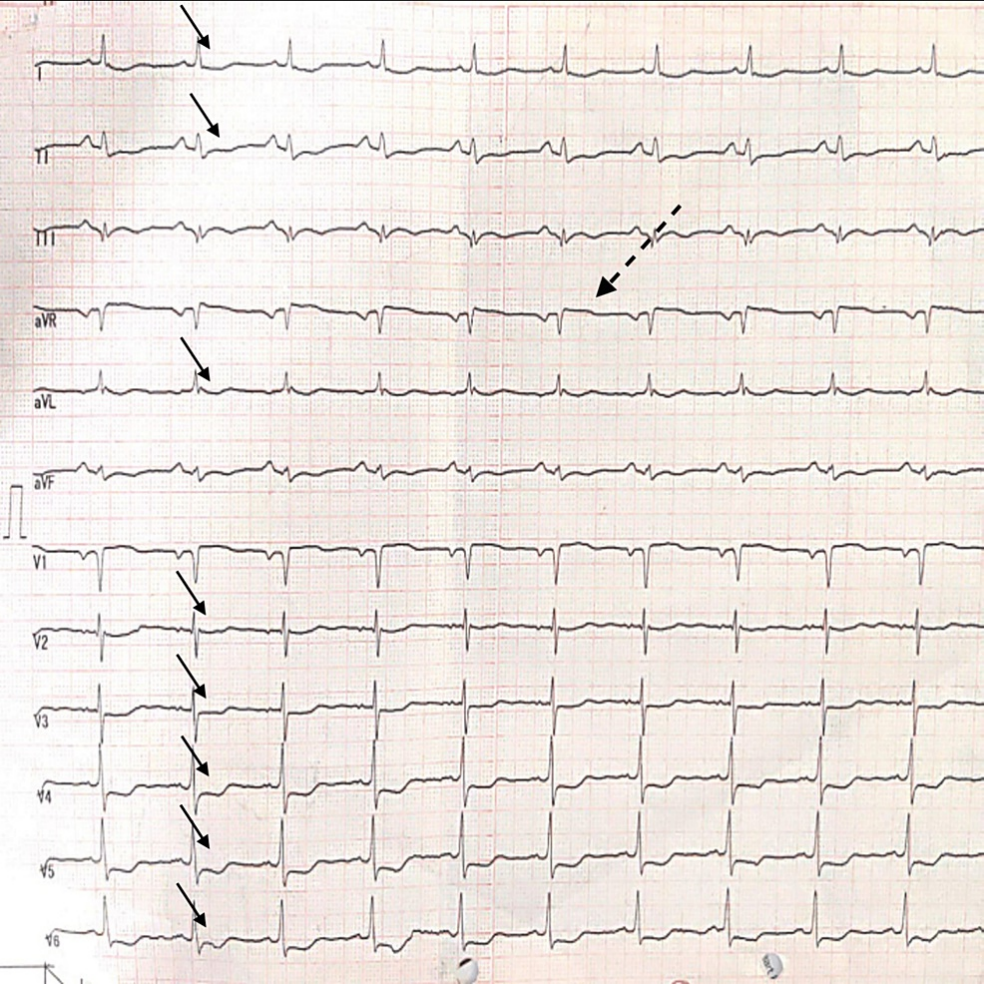
ST elevation in lead aVR and ST depression in leads V2-V6, leads I, II, and aVL The dotted arrow shows ST elevation in lead aVR and and the regular arrows show ST depression in leads V3-V6, leads I, II, and aVL.

On admission, the ECG revealed depression in ST segment in precordial leads V2-V6, as well as limb leads like I, II, and aVL and elevation of 1 mm in ST segment in the aVR (Figure 1). Troponin I level was 42,150 ng/mL (normal range: 1.5-30,000 ng/mL). The first diagnosis was non-ST-elevation myocardial infarction (NSTEMI). 

Because there were ECG evidence of ischemia globally (depression in ST segment in six leads and elevation in ST segment in aVR) and a typical pattern of ECG suggesting significant coronary artery disease, including stenosis in LMCA and the ST-elevation myocardial infarction, urgent coronary angiography was recommended. Clopidogrel 300 mg, acetylsalicylic acid 300 mg, and rosuvastatin 80 mg were given to the patient. After that, the patient was taken straight to the catheterization lab.

On angiography, the LMCA and collateral circulation were entirely blocked. The LMCA was opened by thrombus aspiration and stent placement. A tiny left circumflex coronary artery was discovered after the LMCA was opened, and the left anterior descending (LAD) was 80 percent stenosed (Video 1). After reperfusion, an ECG revealed a sinus rhythm of 68 beats per minute and the ST-segment abnormalities had resolved. On the eighth day of her hospital stay, the patient made a full recovery and was discharged home.

 

**Video 1:** Coronary angiography showing total occlusion of the left main coronary artery (LMCA) The video shows coronary angiography: the first arrow shows thrombotic lesion in LMCA and the later arrow shows left circumflex which is occluded from ostium.

## Discussion

As LMCA branches into the LAD and left circumflex arteries, the LMCA delivers blood to the majority of the left ventricle's anterolateral and septal areas. A low flow of blood following LMCA narrowing affects a major portion of the heart, creating electrical abnormalities visible on the ECG, due to ischemia in myocardium. LMCA or triple vessel stenosis-like patterns have previously been characterized as isolated ST-segment elevation in lead aVR, as well as global ST-segment depression with/without elevation in ST segment in aVR [1-3].

Because the positive end of lead aVR leads to the patient's right shoulder, occlusion of the LMCA will also affect the branches of the LAD that supply septum. The LMCA obstruction is considered to generate basal septum ischemia/injury, leading in a right superior pointing damage vector, which causes ST-segment elevation in aVR lead [4]. Because aVR lead is pointing towards left ventricle, the same electrical vector that causes ST depression in leads V5 and V6 (which are directed towards the apex) will be recorded by aVR lead as ST elevation [5]. When the left ventricular end diastolic pressure is increased, these reciprocal alterations can be detected.

As recommended by the AHA, an ECG showing depression in ST segment of more than 0.1 mV in eight or more leads and elevation in ST segment in lead aVR such that the automated interpretation should suggest that “ischemia occurring because of left main coronary artery or three-vessel coronary artery obstruction”[1]. We obviously cannot add symptoms or risk factors to the computerized automatic interpretation at the moment. As a result, current guidelines indicate that the ECG be interpreted regardless of the clinical presentation. We cannot rule out the potential that in some of our patients, diseases of small vessel or a supply/demand imbalance causes generalized ischemia of subendocardium. Other medical problems, such as hypothermia, have previously been recorded [6] and various neurological disorders [7] may present with such ECG changes transiently. As a result, Samuel Sclarovsky's term "circumferential subendocardial ischemia" or "circumferential subendocardial stress" may be preferable to "ischemia owing to multivessel or left main coronary artery occlusion" [1].

Gorgels et al. were the first to show that aVR ST elevation combined with ST depression in leads I, II, and V4-V6 were indicative of LMCA or three-vessel disease, especially when the total magnitude of ST alterations surpassed 12 mm [8]. ST elevation in lead aVR (N0.05 mV) larger than ST elevation in lead V1 "distinguished" LMCA obstruction from LAD and right coronary artery occlusion, according to Yamaji et al. [9].

This study was done in rural setup where early diagnosis is important to avoid mortality and morbidity. In spite of abundant literature available on this subject, awareness amongst rural hospital is still not there. Hence, this study has been done from rural setup.

## Conclusions

Diagnosis the serious life threatening condition using primitive investigation like ECG is important for physician as early detection of the LMCA blockage or three-vessel coronary artery disease can lead to more effective treatment and reduced morbidity. The patient was identified with LMCA blockage in this case and was treated promptly. It is important in rural setup where early diagnosis is essential to avoid mortality and morbidity.
